# Depression and Osteoporosis: A Mendelian Randomization Study

**DOI:** 10.1007/s00223-021-00886-5

**Published:** 2021-07-14

**Authors:** Bin He, Qiong Lyu, Lifeng Yin, Muzi Zhang, Zhengxue Quan, Yunsheng Ou

**Affiliations:** 1 Department of Orthopedics, The First Affiliated Hospital of Chongqing Medical University, No. 1 Yi Xue Yuan Road, Yuzhong District, Chongqing, 400016 China; 2 Department of General Practice, The First Affiliated Hospital of Chongqing Medical University, No. 1 Yi Xue Yuan Road, Yuzhong District, Chongqing, 400016 China

**Keywords:** Depression, Osteoporosis, Fracture, Mendelian randomization study

## Abstract

Observational studies suggest a link between depression and osteoporosis, but these may be subject to confounding and reverse causality. In this two-sample Mendelian randomization analysis, we included the large meta-analysis of genome-wide association studies for depression among 807,553 individuals (246,363 cases and 561,190 controls) of European descent, the large meta-analysis to identify genetic variants associated with femoral neck bone mineral density (FN-BMD), forearm BMD (FA-BMD) and lumbar spine BMD (LS-BMD) among 53,236 individuals of European ancestry, and the GWAS summary data of heel BMD (HE-BMD) and fracture among 426,824 individuals of European ancestry. The results revealed that genetic predisposition towards depression showed no causal effect on FA-BMD (beta-estimate: 0.091, 95% confidence interval [CI] − 0.088 to 0.269, SE:0.091, *P* value = 0.320), FN-BMD (beta-estimate: 0.066, 95% CI − 0.016 to 0.148, SE:0.042, *P* value = 0.113), LS-BMD (beta-estimate: 0.074, 95% CI − 0.029 to 0.177, SE:0.052, *P* value = 0.159), HE-BMD (beta-estimate: 0.009, 95% CI − 0.043 to 0.061, SE:0.027, *P* value = 0.727), or fracture (beta-estimate: 0.008, 95% CI − 0.071 to 0.087, SE:0.041, *P* value = 0.844). These results were also confirmed by multiple sensitivity analyses. Contrary to the findings of observational studies, our results do not reveal a causal role of depression in osteoporosis or fracture.

**Supplementary Information:**

The online version contains supplementary material available at 10.1007/s00223-021-00886-5.

## Introduction

The United Nations have predicted that the ratio of people aged more than 65 years to those aged 15–64 years will triple globally by 2100 [1]. Disordered musculoskeletal conditions may result in severe pain and physical disability, and their prevalence will increase as the ageing of society [2]. Among the diseases associated with musculoskeletal conditions, osteoporosis is a common, aging-related disease characterized by decreased bone mineral density (BMD) and increased risk of fracture [3–6]. The treatment of osteoporosis is still a big challenge and growing public health problem in the world [7–9]. Genome-wide association studies (GWASs) has demonstrated that BMD is a highly polygenic trait [10–12].

Depression is the leading cause of disability and one in six people is estimated to develop depression during their lifetime [13]. Depression is a chronic disease that affects 18% of men and 26% of women [14]. Several meta-analyses included cross-sectional or case–control studies to investigate the association of depression and osteoporosis, and found that depression might be a significant risk factor for low BMD and fracture, but the results were not consistent [15–18]. However, none of these studies assessed the their association in the prospective cohort design, and these studies were limited by confounding factors and reverse causality.

Genetic epidemiology has emerged as an important approach to unravel the determinants of diseases, because the inheritance of genetic variants at conception is random and cannot be confounded by other risk factors. Mendelian randomization (MR) study has become an effective, powerful and efficient method to establish the causal relationships between exposure phenotype and exposure phenotype through using the GWAS summary statistics [19, 20]. These genetic variants in GWAS summary statistics are randomly allocated before birth and fixed at conception, and thus serve as unconfounded proxies for modifiable risk factors, which affords an analogy to randomized controlled trials (RCTs) in a non-experimental (observational) setting [21, 22].

Two-sample MR analysis greatly increases the scope and statistical power of MR using the published summary data from GWASs [23, 24]. In this study, we use single nucleotide polymorphisms (SNPs) strongly associated with depression as instrumental variables. To our knowledge, this is the first two sample MR study to explore the causal effect of depression on forearm BMD (FA-BMD), femoral neck BMD (FN-BMD), lumbar spine BMD (LS-BMD), heel BMD (HE-BMD) and fracture.

## Methods

### Data on Depression

A large GWAS meta-analysis of depression involved 807,553 individuals of European ancestry (246,363 cases and 561,190 controls) from the three largest GWASs [13]. Depression was defined as a debilitating psychiatric illness that was typically associated with low mood and anhedonia. Initially, 102 independent SNPs were identified to have robust association with depression at the GWAS threshold of statistical significance (*P* < 5 × 10^–8^) after adjusting for sex and age (Supplementary Table 1). These SNPs involved both genes and gene-pathways associated with synaptic structure and neurotransmission.

In one MR study, SNPs were ideally expected to not be in linkage disequilibrium (LD), because SNPs in strong LD may produce some bias. We performed the clumping process (*R*^2^ < 0.001, window size = 10,000 kb) with the European samples from the 1000 genomes project and estimated LD between SNPs. Among the pairs of SNPs with *r*^2^ ≥ 0.001, the SNP with a larger association *P* value would be removed. We also excluded the SNPs that were absent from the LD reference panel. Therefore, 23 SNPs were excluded due to LD and 79 SNPs remained for the subsequent analysis. Finally, 78 SNPs for FA-BMD, 79 SNPs for FN-BMD and LS-BMD, 74 SNPs for HE-BMD and fracture were used as the instrumental variables (Table 1).

**Table 1:** Summary genetic instruments between depression and different outcomes

SNPs	Beta	SE	*P* value	Beta	SE	*P* value	Beta	SE	*P* value	Beta	SE	*P* value	Beta	SE	*P* value	Beta	SE	*P* value
	Depression			FA-BMD			FN-BMD			LS-BMD			HE-BMD			Fracture		
rs301799	− 0.025	0.0035	1.36E-12	− 0.0264	0.01593	0.1036	− 0.0203	0.00757	0.00866	− 0.0239	0.00883	0.00835	− 0.0246	0.00186	4.80E−34	− 0.0026	0.00665	0.7
rs1002656	− 0.0266	0.0038	3.74E-12	0.02817	0.01683	0.10079	− 0.0035	0.00816	0.67805	− 0.0126	0.00949	0.19541	0.00063	0.00202	0.38	0.00229	0.00721	0.74
rs1466887	− 0.0199	0.0036	4.12E-08	− 0.0256	0.01561	0.10816	− 0.0107	0.00758	0.16809	− 0.0084	0.00883	0.35019	− 0.0004	0.00185	0.96	0.00042	0.00662	1
rs11579246	0.0381	0.0061	5.71E-10	0.00879	0.0267	0.74675	− 0.0082	0.01278	0.52944	0.00323	0.01487	0.8321	0.00045	0.00323	0.75	− 0.0026	0.01155	0.84
rs1890946	− 0.0235	0.0035	2.68E-11	0.0037	0.01568	0.81703	− 0.0112	0.00751	0.14335	− 0.0196	0.00877	0.02921	− 0.001	0.00184	0.8	− 6E-06	0.00657	0.96
rs10789214	0.0193	0.0035	4.44E-08	0.00242	0.01606	0.88267	− 0.0104	0.00763	0.18399	− 0.0181	0.00894	0.04838	− 0.0135	0.00185	4.00E−09	0.0273	0.00661	3.60E−05
rs2568958	0.0373	0.0036	8.47E-25	0.01024	0.01613	0.53362	0.00867	0.00774	0.27272	− 0.0047	0.00902	0.61	0.00953	0.00187	0.00013	− 0.0017	0.00667	0.93
rs113188507	0.0221	0.0039	1.87E-08	0.01278	0.01745	0.47283	− 0.0047	0.00837	0.58383	− 0.0117	0.00978	0.24412	− 0.0064	0.00201	0.004	0.00753	0.00716	0.28
rs10913112	− 0.0264	0.0036	3.4E-13	− 0.0225	0.01605	0.16909	− 0.002	0.00779	0.80043	− 0.0117	0.00909	0.20807	0.00537	0.00189	0.0038	0.0032	0.00675	0.69
rs17641524	− 0.032	0.0043	1.52E-13	− 0.0151	0.01922	0.44162	− 0.0031	0.00909	0.74048	− 0.0008	0.01052	0.9424	− 0.0025	0.00225	0.53	− 0.0114	0.00803	0.16
rs12052908	− 0.022	0.0035	4.44E-10	− 0.0107	0.01589	0.50867	− 0.0039	0.00774	0.62409	− 0.0015	0.009	0.86921	0.00326	0.00185	0.27	− 0.0061	0.0066	0.37
rs1568452	0.0248	0.0036	8.12E-12	1.7E-05	0.01606	0.99917	0.01164	0.00777	0.14268	0.00475	0.00905	0.60875	0.00608	0.00189	0.048	0.00602	0.00674	0.33
rs7585722	− 0.0269	0.0048	2.68E-08	− 0.0153	0.02156	0.48689	0.01297	0.01023	0.21485	0.00564	0.01191	0.64353	− 0.0034	0.00254	0.19	− 0.0046	0.00909	0.61
rs1226412	0.0256	0.0043	3.46E-09	0.02059	0.01899	0.28758	− 0.0003	0.00912	0.97429	− 0.004	0.01064	0.71156	− 0.0019	0.00227	0.69	− 0.0036	0.00813	0.68
rs62188629	0.0236	0.0038	7.13E-10	− 0.0099	0.01664	0.56011	0.00916	0.0081	0.2686	0.0056	0.00941	0.5612	0.00179	0.00198	0.41	0.0012	0.00707	0.82
rs4346585	− 0.0236	0.0038	7.13E-10	0.02361	0.01758	0.18785	− 0.0015	0.00892	0.86657	− 0.0012	0.00974	0.90609	_	_	_	_	_	_
rs141954845	0.0229	0.0037	8.15E-10	− 0.0493	0.02504	0.05352	− 0.009	0.01172	0.45135	− 0.0141	0.01295	0.28695	_	_	_	_	_	_
rs6783233	0.0218	0.0039	2.9E-08	− 0.0236	0.01737	0.18258	− 0.0099	0.00838	0.24801	− 0.018	0.00981	0.07242	0.00086	0.00205	0.82	− 0.0101	0.00726	0.16
rs1095626	− 0.0264	0.0035	7.13E-14	0.00375	0.01646	0.8234	− 0.0026	0.00771	0.74523	− 0.0043	0.00903	0.64485	− 0.0015	0.00187	0.27	− 0.0008	0.00663	0.93
rs7685686	0.0202	0.0036	2.57E-08	0.00327	0.01606	0.84188	− 0.0057	0.00762	0.46244	− 0.0017	0.00887	0.84855	− 0.0008	0.00187	0.98	− 0.0072	0.00663	0.31
rs34937911	0.0304	0.0055	4.13E-08	0.02452	0.02456	0.32768	0.00712	0.01165	0.55029	0.0031	0.01355	0.82324	0.0015	0.0029	0.93	0.00115	0.01029	0.96
rs45510091	0.0448	0.008	1.83E-08	0.01463	0.03488	0.68083	0.00875	0.01728	0.62017	− 0.0249	0.02009	0.22638	− 0.0015	0.00403	0.28	− 0.0003	0.01428	0.95
rs35553410	− 0.0244	0.004	1.42E-09	0.02599	0.01766	0.149	0.00032	0.00862	0.97139	− 0.0118	0.01002	0.25058	− 0.0034	0.00211	0.069	0.01234	0.00749	0.096
rs7659414	− 0.0201	0.0035	1.2E-08	0.02273	0.01567	0.15501	0.01486	0.00757	0.05482	0.00727	0.00882	0.42061	− 0.0022	0.00187	0.27	− 0.0005	0.00662	0.92
rs60157091	0.02	0.0035	1.42E-08	− 0.0065	0.01599	0.69031	− 0.0029	0.00764	0.70746	− 0.018	0.00892	0.04935	− 0.0054	0.00185	0.0016	0.01408	0.00654	0.029
rs3099439	− 0.0276	0.0035	5.05E-15	0.0282	0.01588	0.08158	0.00137	0.00875	0.87804	− 0.0144	0.00938	0.13467	− 0.0014	0.00187	0.43	0.00313	0.0066	0.63
rs10061069	− 0.0275	0.0042	8.15E-11	− 0.0292	0.01875	0.12668	− 0.0083	0.00906	0.36817	− 0.0074	0.01055	0.49125	− 0.0023	0.00221	0.16	− 0.001	0.00784	0.95
rs30266	0.0308	0.0037	1.45E-16	0.01996	0.01655	0.23684	− 0.0098	0.008	0.23013	− 0.0063	0.00931	0.50588	− 0.0024	0.00197	0.04	0.02085	0.00697	0.0035
rs11135349	− 0.0295	0.0035	6.04E-17	− 0.0502	0.01596	0.00204	− 0.0083	0.00758	0.28481	− 0.0113	0.00883	0.21168	− 0.0016	0.00185	0.087	− 0.0006	0.00655	0.96
rs200949	0.048	0.0053	2.53E-19	0.05445	0.02691	0.04732	− 0.0074	0.01191	0.544	0.0106	0.01431	0.46792	− 0.0155	0.00261	8.40E-09	0.00722	0.00932	0.47
rs9363467	0.0237	0.0036	6.44E-11	0.00949	0.01679	0.57962	0.00055	0.00795	0.94645	0.00542	0.00923	0.56652	_	_	_	_	_	_
rs7758630	− 0.0225	0.0036	5.56E-10	− 0.0237	0.01616	0.15097	− 0.0009	0.00774	0.90833	− 0.0095	0.00905	0.30343	− 0.0027	0.00187	0.18	0.01485	0.00667	0.017
rs2876520	− 0.023	0.0036	2.29E-10	0.01863	0.01586	0.24951	0.00354	0.00764	0.65027	− 0.0072	0.0089	0.4267	− 0.0024	0.00185	0.28	− 0.0022	0.00662	0.76
rs725616	0.0204	0.0036	1.87E-08	0.01789	0.01632	0.2825	0.01028	0.00777	0.19568	0.02325	0.00907	0.01232	0.0046	0.0019	0.026	− 0.0023	0.00681	0.78
rs2029865	− 0.0201	0.0035	1.2E-08	0.00015	0.01588	0.99252	0.0006	0.00754	0.93839	0.01312	0.00879	0.14466	− 0.0004	0.00184	0.69	0.01112	0.00657	0.088
rs3823624	0.0272	0.0045	1.99E-09	− 0.0037	0.0198	0.85312	− 0.0027	0.00954	0.7833	− 0.0016	0.01109	0.88677	− 0.0073	0.00238	0.0027	0.00413	0.00855	0.61
rs2043539	0.0273	0.0035	9.89E-15	− 0.0152	0.01598	0.35191	0.00737	0.0076	0.34314	0.01577	0.00885	0.08185	0.0047	0.00185	0.039	− 0.0022	0.00662	0.77
rs2247523	− 0.0207	0.0035	4.38E-09	0.02397	0.01573	0.13522	0.01087	0.00753	0.1582	0.00152	0.0088	0.8661	0.00196	0.00183	0.65	0.01039	0.00656	0.1
rs58104186	0.0237	0.0035	1.82E-11	0.01034	0.0158	0.52106	0.01124	0.00751	0.14328	0.01003	0.00877	0.26397	0.00265	0.00184	0.19	− 0.0112	0.0066	0.092
rs7837935	− 0.0292	0.0049	3.34E-09	− 0.0538	0.0218	0.01555	− 0.0065	0.01053	0.54684	− 0.0124	0.01252	0.33492	0.00076	0.00253	0.72	0.00744	0.00894	0.42
rs67436663	− 0.0259	0.0042	9.37E-10	− 0.0365	0.035	0.30658	0.00566	0.01649	0.73723	− 0.0329	0.01813	0.07657	_	_	_	_	_	_
rs1354115	0.021	0.0036	7.08E-09	− 0.0182	0.0247	0.46894	0.01734	0.00932	0.06883	0.02748	0.01119	0.01646	− 0.0052	0.00192	0.045	0.00041	0.00678	0.92
rs263645	0.0221	0.0035	3.7E-10	0.01283	0.01557	0.41884	− 0.0096	0.0075	0.21017	0.00035	0.00872	0.96841	− 0.001	0.00186	0.61	− 0.0122	0.00658	0.083
rs34653192	− 0.0229	0.0038	2.23E-09	0.00931	0.01685	0.01685	0.00756	0.00812	0.36268	0.00661	0.00947	0.4952	0.00246	0.002	0.061	− 0.0085	0.00708	0.2
rs10817969	0.0261	0.0039	3.11E-11	_	_	_	− 0.0099	0.01554	0.53372	0.00554	0.01904	0.77542	− 0.0032	0.00206	0.15	− 0.0065	0.00728	0.35
rs997934	0.0198	0.0036	4.81E-08	− 0.007	0.01585	0.66476	0.00572	0.00771	0.46745	0.00558	0.00896	0.54274	− 0.0007	0.0019	0.4	− 0.0078	0.00675	0.25
rs1021363	0.0303	0.0037	4.41E-16	0.02478	0.0161	0.13132	− 0.0067	0.00781	0.40034	− 0.002	0.00909	0.83214	− 0.0026	0.00192	0.2	0.0101	0.00683	0.14
rs1448938	0.0214	0.0035	1.3E-09	0.00077	0.01591	0.96215	0.01071	0.00763	0.16948	0.03221	0.00889	0.00041	0.0087	0.00186	4.50E-05	− 0.0218	0.00662	0.0011
rs2509805	0.022	0.0038	9.17E-09	0.02034	0.01704	0.24191	0.02674	0.00869	0.00263	0.00512	0.0095	0.59892	0.00953	0.00198	0.00024	− 0.0055	0.00702	0.4
rs198457	− 0.0292	0.0046	2.99E-10	− 0.0022	0.02051	0.91518	− 0.0026	0.00969	0.79206	− 0.0026	0.01132	0.8199	− 0.0094	0.00237	6.00E-04	0.01139	0.00843	0.18
rs7932640	0.0281	0.0035	1.62E-15	− 0.017	0.01605	0.29952	− 0.0025	0.00762	0.75202	− 0.0013	0.00887	0.88201	0.00791	0.00186	6.00E-04	0.00057	0.00663	0.93
rs61902811	− 0.0257	0.0036	1.4E-12	− 0.0233	0.01594	0.15229	− 0.0202	0.0077	0.01031	− 0.0183	0.00895	0.04618	0.00341	0.00191	0.069	− 0.0011	0.00678	0.88
rs57344483	− 0.038	0.0068	1.82E-08	− 0.0227	0.02863	0.43741	− 0.034	0.01404	0.01404	− 0.014	0.01626	0.40155	0.00054	0.00349	0.47	0.015	0.01241	0.18
rs78337797	0.0306	0.0055	3.37E-08	0.00291	0.02436	0.90687	0.002	0.0121	0.87131	0.01798	0.01409	0.21268	0.00024	0.00283	0.66	− 0.0163	0.01002	0.11
rs56314503	− 0.0254	0.004	2.95E-10	0.00461	0.01848	0.80687	− 0.0128	0.0087	0.14904	− 0.0004	0.01012	0.96604	− 0.0004	0.0022	0.74	− 0.0065	0.00781	0.45
rs10774600	− 0.0267	0.0048	3.39E-08	− 0.015	0.02151	0.49556	− 0.0076	0.01027	0.46869	− 0.0019	0.01198	0.87633	− 0.0022	0.00254	0.58	− 0.0175	0.00898	0.05
rs3213572	0.0217	0.0035	7.61E-10	0.0188	0.01599	0.24878	− 0.0005	0.0076	0.95115	0.00525	0.00886	0.56296	− 0.0056	0.00185	0.0067	0.00642	0.00655	0.29
rs1409379	0.0249	0.0041	1.67E-09	− 0.046	0.01822	0.01325	− 0.0009	0.00885	0.91719	0.00369	0.01035	0.72778	− 0.0104	0.00218	1.10E-05	0.00443	0.0077	0.57
rs1343605	0.0313	0.0036	6.23E-18	0.01126	0.01629	0.49772	− 0.0117	0.00777	0.14229	− 0.0149	0.00909	0.10983	− 0.0055	0.00192	0.00054	0.00316	0.00678	0.57
rs9592461	0.0216	0.0035	9.1E-10	− 0.0251	0.01568	0.11596	0.00195	0.00746	0.79799	− 0.0038	0.0087	0.67332	0.00186	0.00186	0.26	0.00784	0.00654	0.29
rs9545360	− 0.0271	0.0046	5.02E-09	0.00039	0.02007	0.98481	− 0.0062	0.0097	0.53208	− 0.0115	0.01126	0.31934	0.00137	0.0024	0.76	0.00857	0.00847	0.35
rs4772087	0.0227	0.0036	3.91E-10	− 0.0068	0.01605	0.67583	− 0.0088	0.00827	0.29778	− 0.0031	0.00903	0.7377	− 0.0039	0.00192	0.11	− 0.0059	0.00676	0.37
rs61990288	− 0.026	0.0035	1.68E-13	0.02428	0.01566	0.12827	0.00424	0.00752	0.58132	0.01666	0.00877	0.06347	0.00265	0.00186	0.37	0.00867	0.00655	0.21
rs1956373	− 0.0226	0.004	2.06E-08	0.00262	0.01809	0.88722	0.01594	0.00859	0.06935	0.01084	0.01001	0.29047	− 0.0001	0.00215	0.69	0.00874	0.00757	0.26
rs1045430	− 0.0253	0.0035	7.31E-13	− 0.017	0.01574	0.01574	− 0.0024	0.00752	0.75718	− 0.0006	0.0087	0.94807	− 0.0061	0.00185	0.0012	0.00033	0.00653	0.96
rs10149470	− 0.0267	0.0035	3.72E-14	0.00896	0.01552	0.01552	0.00388	0.00757	0.61581	0.01119	0.00882	0.21519	0.0036	0.00186	0.11	− 0.0003	0.00655	0.98
rs8037355	− 0.0233	0.0035	3.94E-11	− 0.0221	0.01567	0.1673	0.00688	0.00756	0.37297	− 0.0216	0.00882	0.01694	0.00159	0.00186	0.46	− 0.0197	0.00804	0.014
rs34488670	− 0.0252	0.0043	6.03E-09	0.01255	0.0193	0.52388	− 0.0068	0.00916	0.46635	0.00959	0.0107	0.38127	0.00116	0.00228	0.7	0.00697	0.00706	0.32
rs7193263	− 0.0239	0.0038	4.33E-10	0.01443	0.01679	0.39933	− 0.0065	0.00798	0.00798	0.00535	0.00927	0.57319	− 0.0015	0.002	0.75	− 0.0022	0.00678	0.82
rs7198928	0.0239	0.0036	4.45E-11	− 0.0294	0.01566	0.06585	− 0.0202	0.00765	0.00975	− 0.013	0.00889	0.1529	− 0.0033	0.00192	0.065	0.00223	0.00678	0.82
rs12923444	− 0.0214	0.0035	1.3E-09	0.01766	0.01556	0.26598	0.00172	0.00755	0.82395	− 0.0024	0.00879	0.788	0.0063	0.00186	0.00045	− 0.0017	0.00658	0.78
rs75581564	0.0301	0.0054	3.17E-08	− 0.0287	0.02411	0.24268	− 0.0029	0.01181	0.80805	− 0.005	0.01377	0.72061	− 0.0016	0.00283	0.41	0.00388	0.01001	0.71
rs12967855	0.0265	0.0037	1.18E-12	− 0.0164	0.01669	0.33618	0.00124	0.00801	0.87936	− 0.0167	0.00936	0.08202	− 0.0055	0.00198	0.013	0.00095	0.00698	0.89
rs7227069	0.0238	0.0035	1.5E-11	− 0.0047	0.01614	0.7747	0.00273	0.00765	0.72747	− 0.0106	0.00894	0.2479	− 0.0029	0.00188	0.078	0.00769	0.00662	0.24
rs12966052	− 0.0314	0.0046	1.25E-11	− 0.0266	0.02022	0.19715	− 0.0153	0.00973	0.12333	− 0.007	0.01133	0.54609	0.00175	0.00241	0.55	− 0.0054	0.00848	0.52
rs7241572	0.028	0.0044	2.7E-10	0.03054	0.03054	0.12563	0.01589	0.00959	0.10503	0.00952	0.01121	0.40695	0.00637	0.00232	0.0018	0.00917	0.00815	0.23
rs33431	0.0198	0.0036	4.81E-08	0.01177	0.01593	0.46893	0.0009	0.00766	0.90848	− 0.0083	0.0089	0.36478	0.00112	0.00192	0.89	0.01221	0.00677	0.067
rs143186028	0.0277	0.0046	2.29E-09	− 0.0153	0.02044	0.46353	0.00894	0.01062	0.41034	− 0.0048	0.0116	0.68915	_	_	_	_	_	_
rs5995992	− 0.0266	0.0039	1.3E-11	− 0.0215	0.01823	0.24781	− 0.0131	0.01029	0.21159	0.00548	0.01176	0.64907	− 0.0123	0.00206	9.80E-10	0.00483	0.00723	0.5

*SNP* single nucleotide polymorphism, *SE* standard error

### Data on BMD and Fracture

Osteoporotic fractures commonly occur in the skeletal sites including femoral neck, forearm, lumbar spine and heel [25]. One large meta-analysis reported the genetic variants associated with FN-BMD, FA-BMD and LS-BMD among 53,236 individuals of European ancestry. Each SNP was tested after adjusting for sex, age, age^2^ and weight [25]. In addition, the GWAS summary data for the associations with HE-BMD and fracture were obtained from 426,824 individuals of European ancestry after adjusting for age, sex and genotyping [3]. Table 1 presented the genetic associations between depression and each outcome including FN-BMD, FA-BMD, LS-BMD, HE-BMD and fracture.

### Statistical Analyses

To determine MR estimates of depression for FN-BMD, FA-BMD, LS-BMD, HE-BMD and fracture, we conducted the inverse variance-weighted (IVW) meta-analysis of Wald ratio for individual SNPs. The weighted median and MR-Egger regression methods were also applied to estimate the effects. The MR method was based on the following three assumptions: (i) instrumental variables were strongly associated with depression; (ii) instrumental variables affected outcomes only through their effect on depression and not through any alternative causal pathway; and (iii) instrumental variables were independent of any confounders [26]. The strength of each instrument was measured by calculating the F-statistic using the following formula: *F* = *R*^2^(*N*−2)/(1−*R*^2^), where *R*^2^ was the proportion of the depression variability explained by each instrument and *N* was the sample size of the GWAS for the depression association [27].

### Sensitivity Analyses

Several sensitivity analyses were used to check and correct for the presence of pleiotropy in the causal estimates. Cochran’s Q was computed to quantify heterogeneity across the individual causal effects, and the random effects IVW MR analysis was used [28, 29]. To assess the potential violation of these assumptions, we evaluated the directional pleiotropy based on the intercept obtained from the MR-Egger analysis [30]. We also assessed the presence of pleiotropy using the MR pleiotropy residual sum and outlier test (MR-PRESSO), during which outlying SNPs were excluded and the effect estimates were reassessed [31].

All tests were two-tailed, and differences with *P* < 0.05 were considered statistically significant. All of these analyses were conducted in R V.4.0.4 by using R packages of ‘MendelianRandomization’ [32] and ‘TwoSampleMR’ [33].

## Results

### Causal Effect of Depression on FA-BMD, FN-BMD and LS-BMD

**Fig. 1:**
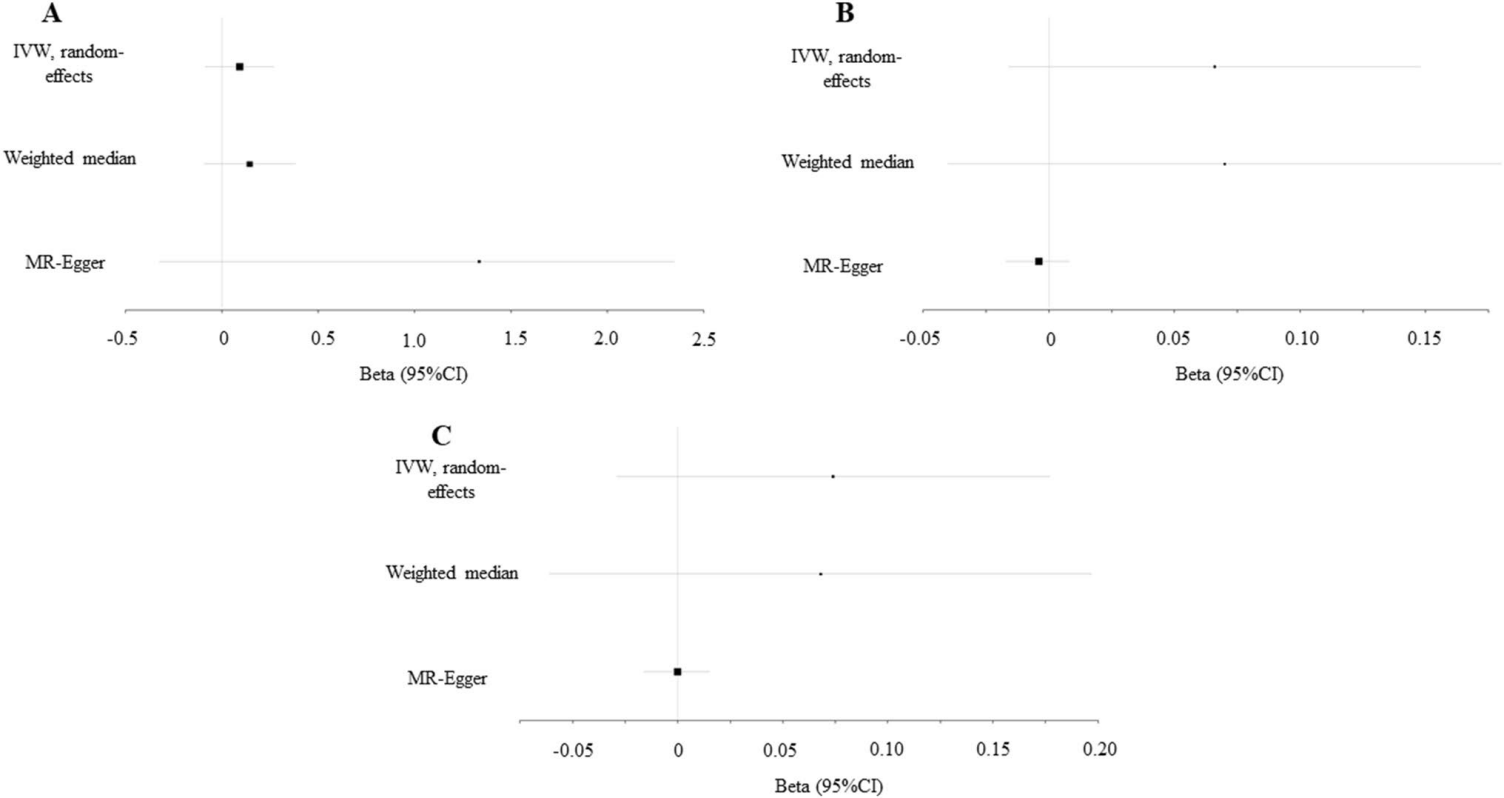
Beta (95% CIs) for association between depression and three sites of BMD (FA-BMD, FN-BMD and LS-BMD). These effects were obtained using summary-level data from the GWASs of depression (*n* = 807,553) on **A** FA-BMD (*n* = 53,236), **B** FN-BMD (*n* = 53,236) and **C** LS-BMD (*n* = 53,236). Error bars represented 95% confidence intervals. All statistical tests were two-sided

We evaluated the causal effect of depression on three sites of BMD (FA-BMD, FN-BMD and LS-BMD, Table 2 and Fig. 1). In the primary IVW analyses, depression showed no MR association with FA-BMD (beta-estimate: 0.091, 95% CI − 0.088 to 0.269, SE:0.091, *P* value = 0.320), FN-BMD (beta-estimate: 0.066, 95% CI − 0.016 to 0.148, SE:0.042, *P* value = 0.113) or LS-BMD (beta-estimate: 0.074, 95% CI − 0.029 to 0.177, SE:0.052, *P* value = 0.159). These results were also confirmed by weighted-median analyses with regards to FA-BMD (beta-estimate: 0.144, 95% CI − 0.092 to 0.380, SE:0.120, *P* value = 0.231), FN-BMD (beta-estimate: 0.070, 95% CI − 0.061 to 0.180, SE:0.056, *P* value = 0.215) or LS-BMD (beta-estimate: 0.068, 95% CI − 0.702 to 0.197, SE:0.066, *P* value = 0.302).

**Table 2:** Mendelian randomization estimates of depression on outcomes

Variables	IVW							Weighted median				MR-Egger							
	Estimate	SE	95% CI	*P* value	*Q* value	*I*^2^	Heterogeneity *P* value	Estimate	SE	95% CI	*P* value	Estimate	SE	95% CI	*P* value	Intercept	SE	95% CI	Pleiotropy *P* value
FA-BMD	0.091	0.091	− 0.088,0.269	0.320	103.801	25.80%	0.023	0.144	0.120	− 0.092,0.380	0.231	1.335	0.516	− 0.324,2.347	0.010	− 0.032	0.013	− 0.058,-0.006	0.014
FN-BMD	0.066	0.042	− 0.016,0.148	0.113	96.598	19.30%	0.075	0.070	0.056	− 0.040,0.180	0.215	0.239	0.243	− 0.237,0.714	0.326	− 0.004	0.006	− 0.017,0.008	0.471
LS-BMD	0.074	0.052	− 0.029,0.177	0.159	112.741	30.80%	0.006	0.068	0.066	− 0.061,0.197	0.302	0.083	0.308	− 0.520,0.686	0.787	0.000	0.008	− 0.016,0.015	0.976
HE-BMD	0.009	0.027	− 0.043,0.061	0.727	600.806	87.80%	< 0.0001	− 0.001	0.017	− 0.034,0.033	0.976	0.038	0.150	− 0.256,0.333	0.798	− 0.001	0.004	− 0.008,0.007	0.844
Fracture	0.008	0.041	− 0.071,0.087	0.844	110.664	34.00%	0.029	0.020	0.049	− 0.075,0.115	0.680	0.122	0.229	− 0.326,0.571	0.593	− 0.003	0.006	− 0.014,0.008	0.612

*FA-BMD* forearm BMD, *FN-BMD* femoral neck BMD, *LS-BMD* lumbar spine BMD, *HE-BMD* heel BMD, *IVW* inverse variance weighted, *SE* standard error, *CI* confidence interval

### Causal Effect of Depression on HE-BMD and Fracture

**Fig. 2:**
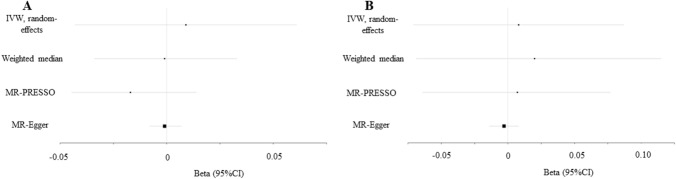
Beta (95% CIs) for association between depression and HE-BMD/fracture. These effects were obtained using summary-level data from the GWASs of depression (*n* = 807,553) on **A** HE-BMD (*n* = 426,824) and **B** fracture (*n* = 426,824). Error bars represented 95% confidence intervals. All statistical tests were two-sided

Depression showed null association with HE-BMD in the IVW (beta-estimate: 0.009, 95% CI − 0.043 to 0.061, SE:0.027, *P* value = 0.727) and weighted-median analyses (beta-estimate: − 0.001, 95% CI − 0.034 to 0.033, SE:0.017, *P* value = 0.976, Table 2 and Fig. 2). Consistently, there was also no relationship between depression and fracture in the results of IVW (beta-estimate: 0.008, 95% CI − 0.071 to 0.087, SE:0.041, *P* value = 0.844) or weighted-median analyses (beta-estimate: 0.020, 95% CI − 0.075 to 0.115, SE:0.049, *P* value = 0.680, Table 2 and Fig. 2).

### Evaluation of Assumptions and Sensitivity Analyses

The strength of the genetic instruments denoted by the *F*-statistic was ≥ 10 for all the depression variants (Supplementary Table 1). Little evidence of directional pleiotropy was found for all models except for FA-BMD (MR-Egger intercept *P* value = 0.014) (Table 2). The estimates from the weighted-median approach for the SNP instrument were all consistent with those of IVW models (Table 2).

Among 79 SNP instrument variables, MR-PRESSO method identified 14 outliers (rs301799, rs10789214, rs2568958, rs1568452, rs200949, rs3823624, rs1448938, rs2509805, rs198457, rs7932640, rs1409379, rs1343605, rs12923444, and rs5995992) for HE-BMD and 2 outliers (rs1448938 and rs10789214) for fracture. After excluding these outliers, depression still revealed no causal effect on HE-BMD (beta-estimate: − 0.017, 95% CI − 0.048 to 0.014, SE:0.016, *P* value = 0.292) or fracture (beta-estimate: 0.007, 95% CI − 0.064 to 0.077, SE:0.036, *P* value = 0.855) (Table 3 and Fig. 2).

**Table 3:** Mendelian randomization estimates between depression and outcomes after sensitivity analysis excluding outlying SNPs detected by MR-PRESSO

Variables	Estimate	SE	95% CI	*P* value
HE-BMD excluding rs301799, rs10789214, rs2568958, rs1568452, rs200949, rs3823624, rs1448938, rs2509805, rs198457, rs7932640, rs1409379, rs1343605, rs12923444, rs5995992	− 0.017	0.016	− 0.048,0.014	0.292
Fracture excluding rs1448938, rs10789214	0.007	0.036	− 0.064,0.077	0.855

*MR-PRESSO* Mendelian randomization pleiotropy residual sum and outlier test, *SNP* single nucleotide polymorphism, *IVW* inverse variance weighted, *SE* standard error, *CI* confidence interval, *HE-BMD* heel BMD

## Discussion

Observational studies reported inconsistent results regarding the association between depression and osteoporosis [34–38]. Positive associations were supported by previous meta-analyses that suggested that depression was associated with low BMD and the increased risk of fracture [15–17, 39, 40]. Previous studies reported that depression may affect bone formation and bone resorption through altering the concentrations of many hormones. For instance, depression elevated the cortisol level through activating the hypothalamic–pituitary–adrenal axis, and hypercortisolemia was an important causal factor to decrease BMD [39]. The inverse regulation between depression and bone formation may be associated with gonadal hormones estrogen, testosterone and growth hormone/insulin growth factors [41, 42].

However, several limitations may result in some bias for these positive results. First, these meta-analyses only included case–control or cross-sectional studies, and it was unclear whether depression was prospectively associated with increased risk of fracture and bone loss. Second, the most of original studies employed self-report scales to define depression, which might produce some bias of misclassification. Third, some original reports lacked the data of medication use such as corticosteroid and glucocorticoid, which may affect the observed association.

This insistent association may be biased due to the methodological limitations (i.e. confounding, reverse causation and measurement error) of traditional observational study [43]. The MR study has been widely used to evaluate causal inferences between risk factors and disease outcomes using genetic variants as instrumental variables [44]. To date, our work is the first two-sample MR study to explore the causal effect of depression on BMD and fracture. Our study included the three large GWASs of depression [13], FA-BMD, FN-BMD and LS-BMD [25], HE-BMD and fracture [3]. This MR analysis revealed no causal effect of depression on four sites of BMD or fracture. We did not find the evidence of a causal link between depression and osteoporosis, which were contrast to previous observational studies. These suggested that false associations between depression and osteoporosis may be caused by confounding factors such as smoking, increased alcohol drinking and decreased physical activity [45].

Several important strengths exit in this study. This is the first two-sample MR study to investigate the causality between depression and osteoporosis, which is the closest approximation to RCT and allows the random allocation based on the genotype. This study design can prevent some limitations of conventional observational studies, including reverse causation and potential confounding factors. The large sample sizes of included studies and instrumental variables robustly associated with depression (*F* statistics ≥ 10) are used in our MR study. The intercepts for the MR-Egger analysis suggest that all observed causal associations are not affected by directional pleiotropy except FA-BMD. We conduct multiple sensitivity analyses to test the influence of pleiotropy on our causal estimates, and our results are robust according to these various tests.

Several limitations also should be taken into consideration. First, all the included participants are of European origin, and more studies should be conducted to confirm whether our findings are generalizable to other populations. Secondly, the broader self-declared definitions of depression are used in the GWAS meta-analysis of depression [13], although there is a strong genetic correlation between broader self-declared definitions of depression and clinically diagnosed depressive disorder [46]. Third, significant heterogeneity remains for the analysis of HE-BMD, which may be caused by different patient population and definitions of depression.

## Conclusion

This two-sample MR provides strong evidence for no casual association between depression and osteoporosis, and suggests the confounding factors and reverse may cause the reported observational associations between them.

## Supplementary Information

Below is the link to the electronic supplementary material.

Supplementary file1 (XLSX 22 kb)

## Data Availability

Data supporting the findings of this study were available within the paper.
